# Supplementary figures and images for: Choline metabolism underpins macrophage IL-4 polarization and RELMα up-regulation in helminth infection
Source: PLoS Pathog. 2023 Sep 25;19(9):e1011658. doi: 10.1371/journal.ppat.1011658 (PMC10553840; doi:10.1371/journal.ppat.1011658)

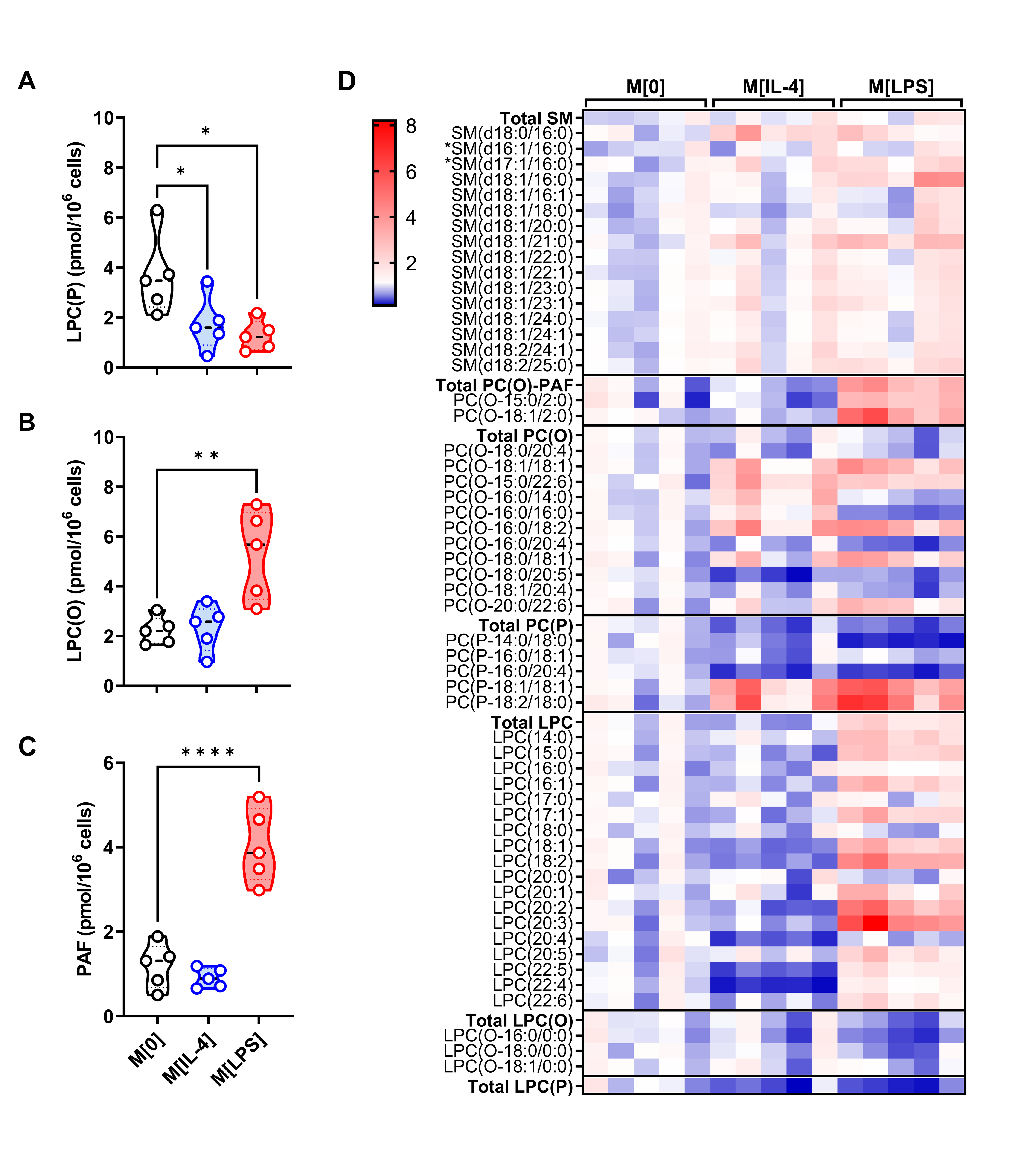

Supplement: S1 Fig — A-I) Sum of phospholipid subclasses. A) Plasmenyl-lysophosphatidylcholines (LPC(P)), B) plasmanyl-LPCs (LPC(O)), C) platelet activating factors (PAF). D) D) Heatmap of sphingomyelin (SM), plasmanyl-PAFs (PC(O)-PAF), PC(O), PC(P), LPC, LPC(O), and LPC(P). Heatmap statistics are shown as fold change over the average of M[0]. n = 5. One-way ANOVA with Dunnett’s test vs M[0] for multiple comparisons (* p < 0.05, ** p < 0.01, **** p < 0.0001). (TIF) [file ppat.1011658.s001.tif]

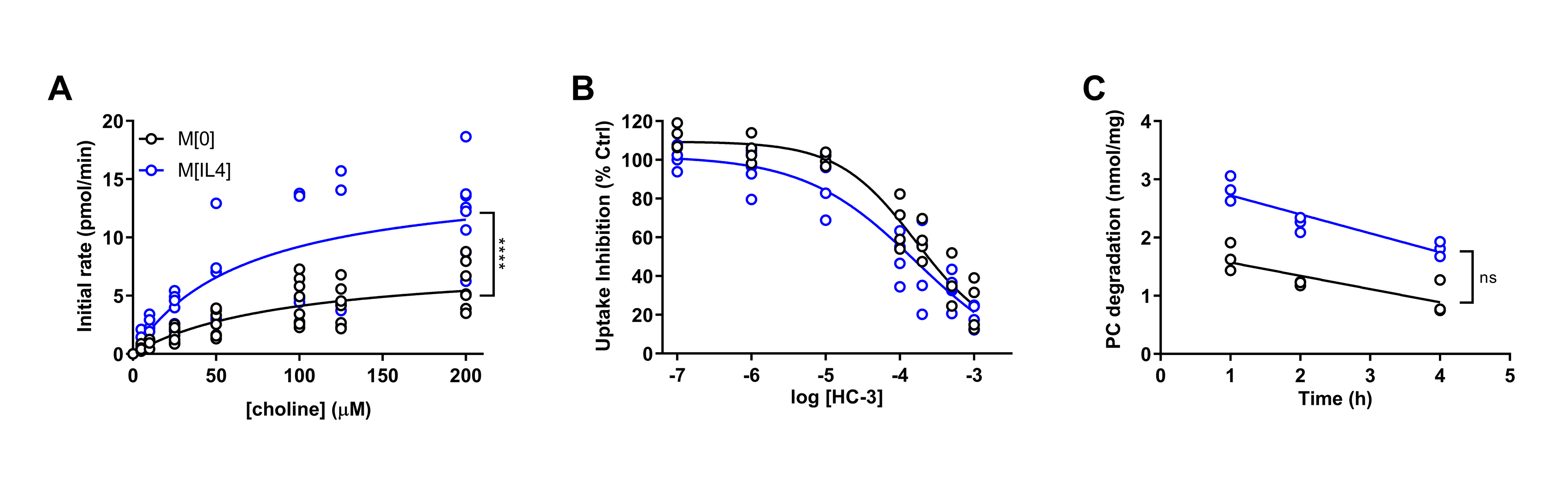

Supplement: S2 Fig — A) Saturation curves showing rate of 3H-choline uptake at increasing concentrations of unlabeled choline. n = 8. Michaelis-Menten least squares fit regression (**** p < 0.0001). B) Inhibition of 3H-choline uptake by HC3. n = 4 (M[0]) or 5 (M[IL-4]). Four parameter log(inhibitor) vs. response regression F test (** p < 0.01). C) Pulse-chase to determine the rate of PC degradation. n = 3. Linear regression with F test for slopes (ns). (TIF) [file ppat.1011658.s002.tif]

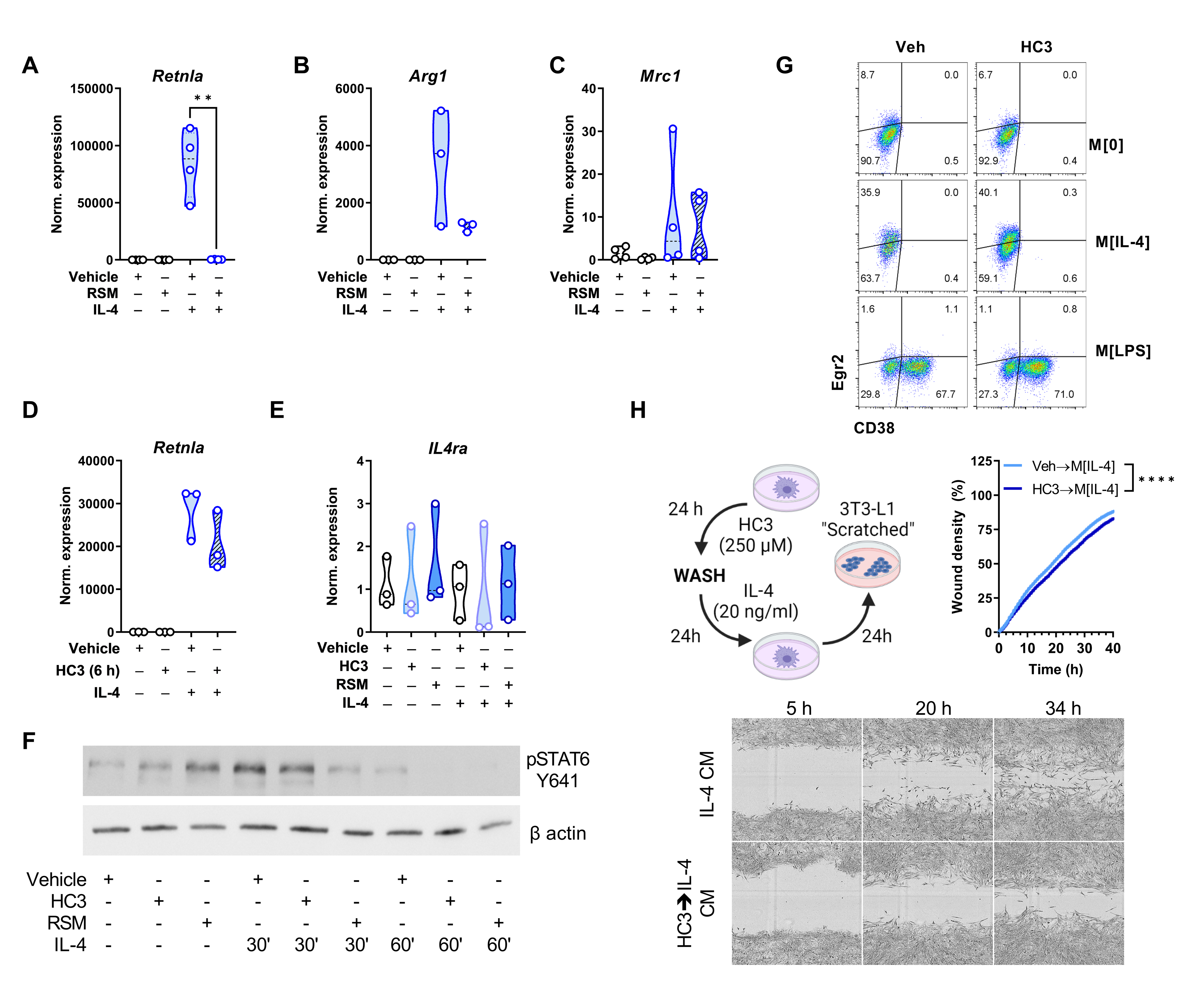

Supplement: S3 Fig — A-C) Macrophages were treated with vehicle (DMSO) or RSM-932a (5 μM) for 24 h, washed, then treated with IL-4 (20 ng/mL) for 24 h. Relative expression of M[IL-4] hallmark genes Retnla, Arg1, Mrc1, normalized to Actb and compared to M[0]. n = 3–4, representative of 3 experiments. Unpaired t test (** p < 0.01). D-E) Macrophages were treated with vehicle (DMSO) or HC3 (250 μM) for 6 h, washed, then treated with IL-4 (20 ng/mL) for 24 h. Relative expression of Retnla or Il4ra (E) normalized to Actb and compared to M[0]. n = 3. F) Macrophages were treated with vehicle (DMSO) or RSM-932a (5 μM) for 24 h, washed, then treated with IL-4 (20 ng/mL). Expression of IL-4 signaling molecule pSTAT6 (Tyr641) compared to β-actin. Representative of n = 5. G) Macrophages were treated with vehicle (DMSO) or HC3 (250 μM) for 24 h, washed, then treated with IL-4 (20 ng/mL) for 24 h. Expression of intracellular Egr2 or surface CD38. n = 3, representative of 2 experiments. H) Left, schematic of wound healing assay with conditioned media. Right, Macrophages were treated with vehicle (DMSO) or HC3 (250 μM) for 24 h, washed, then treated with IL-4 (20 ng/mL) for 24 h and conditioned media was collected. Confluent 3T3-L1 fibroblast monolayers were scratched, and media was replaced with macrophage conditioned media. Images of wound healing over time, quantified by wound density. n = 3, representative of 2 experiments. Sum-of-squares F test of non-linear fit of growth curves (**** p < 0.0001). Lower, snapshots of 3T3-L1 fibroblast wounds at different timepoints. Schematics were created using BioRender. (TIF) [file ppat.1011658.s003.tif]

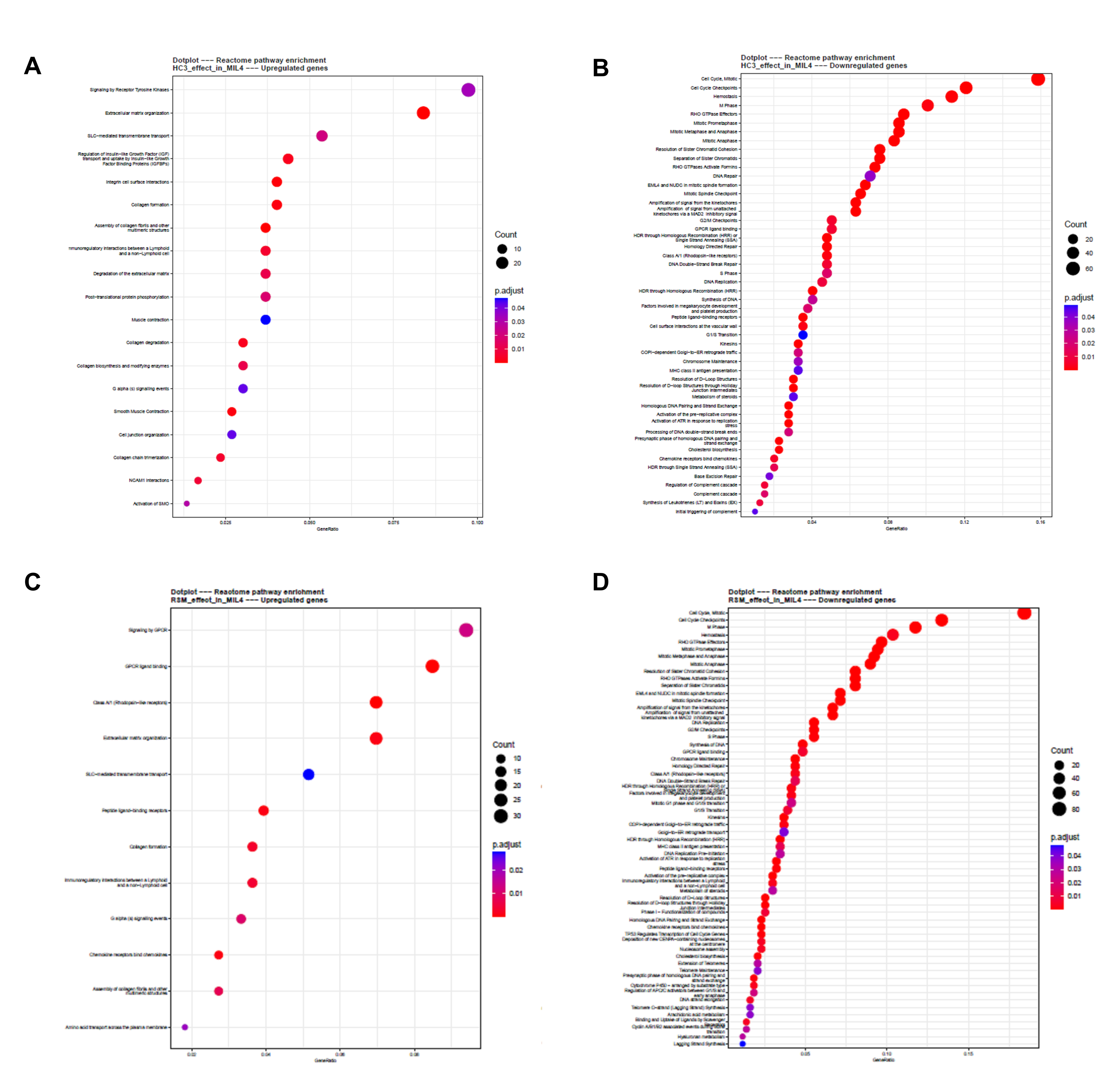

Supplement: S4 Fig — A-D) Reactome pathway analysis of genes up-regulated by HC3 (A) or RSM (C) in M[IL-4] or down-regulated by HC3 (B) or RSM (D), sorted by gene count enrichment for each Reactome pathway. (TIF) [file ppat.1011658.s004.tif]

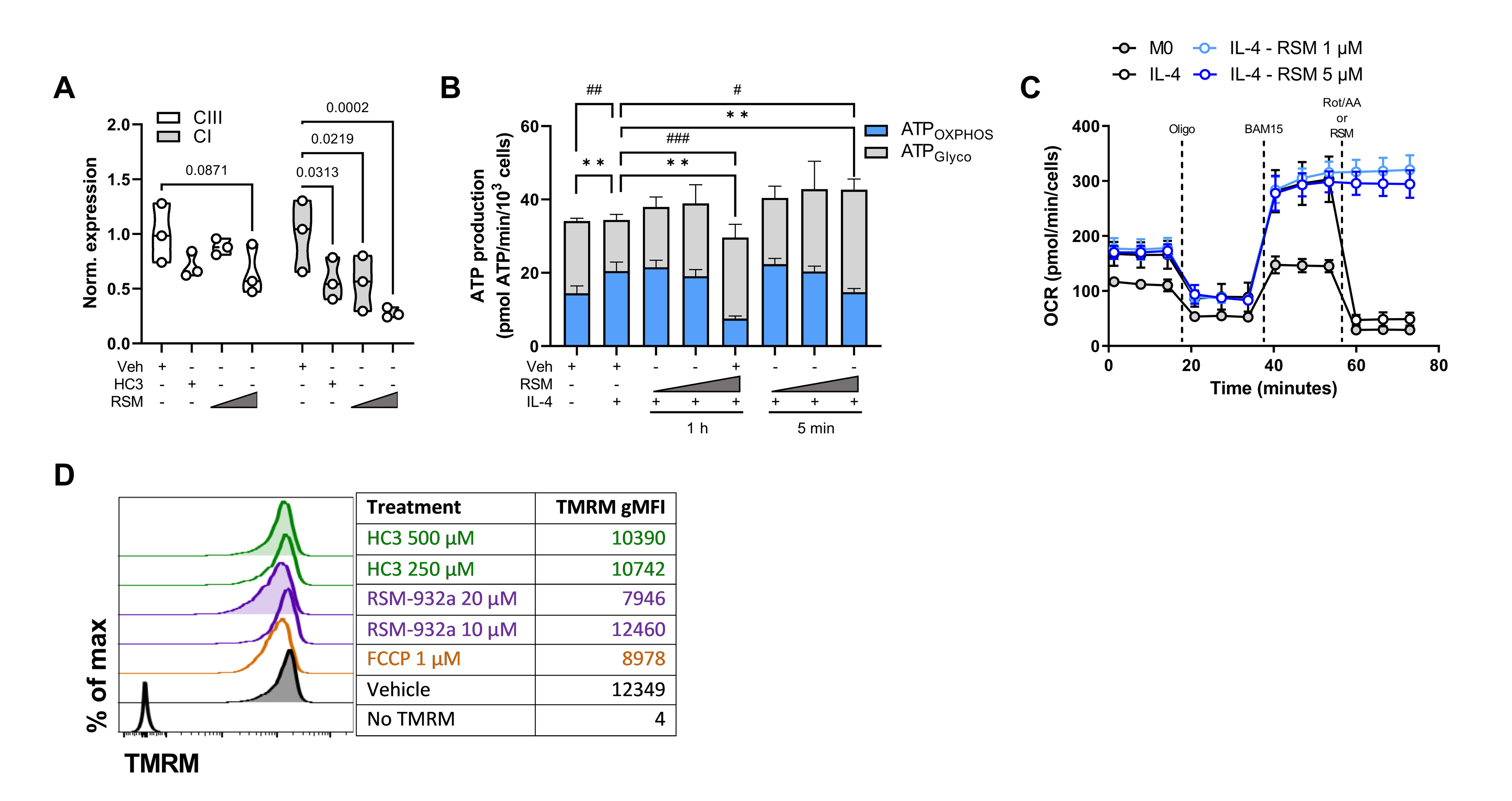

Supplement: S5 Fig — A) Densitometry of complex III and I from Fig 4B. Two-way ANOVA with Dunnett’s test for multiple comparisons. B) Bioenergetics analysis of ATP produced through oxidative phosphorylation (ATPOXPHOS) or glycolysis (ATPGlyco). Macrophages were treated for 1 h or 5 min with RSM (0.2, 1, or 5 μM) prior to Mito Stress Test assay as in Fig 4C–4F. Measurements (n = 3 in triplicate) were normalized per 103 cells. Mixed-effects analysis with Tukey’s test for multiple comparisons (ATPOXPHOS: # p < 0.05, ## p < 0.01, ### p < 0.001; ATPGlyco: ** p < 0.01). C) Mito Stress Test assay of extracellular flux with sequential treatments of 1.5 μM oligomycin, 14 μM BAM15, and 1 μM rotenone/1 μM antimycin A/Hoechst 33342. Oxygen consumption rate (OCR; B) or extracellular acidification rate (ECAR; C) of M[0] or M[IL-4] treated with RSM (1 or 5 μM) in place of rotenone/antimycin A during the last injection. D) Histograms and geometric MFI of tetramethylrhodamine methyl ester (TMRM) staining of macrophages treated with inhibitors or vehicle (DMSO). (TIF) [file ppat.1011658.s005.tif]

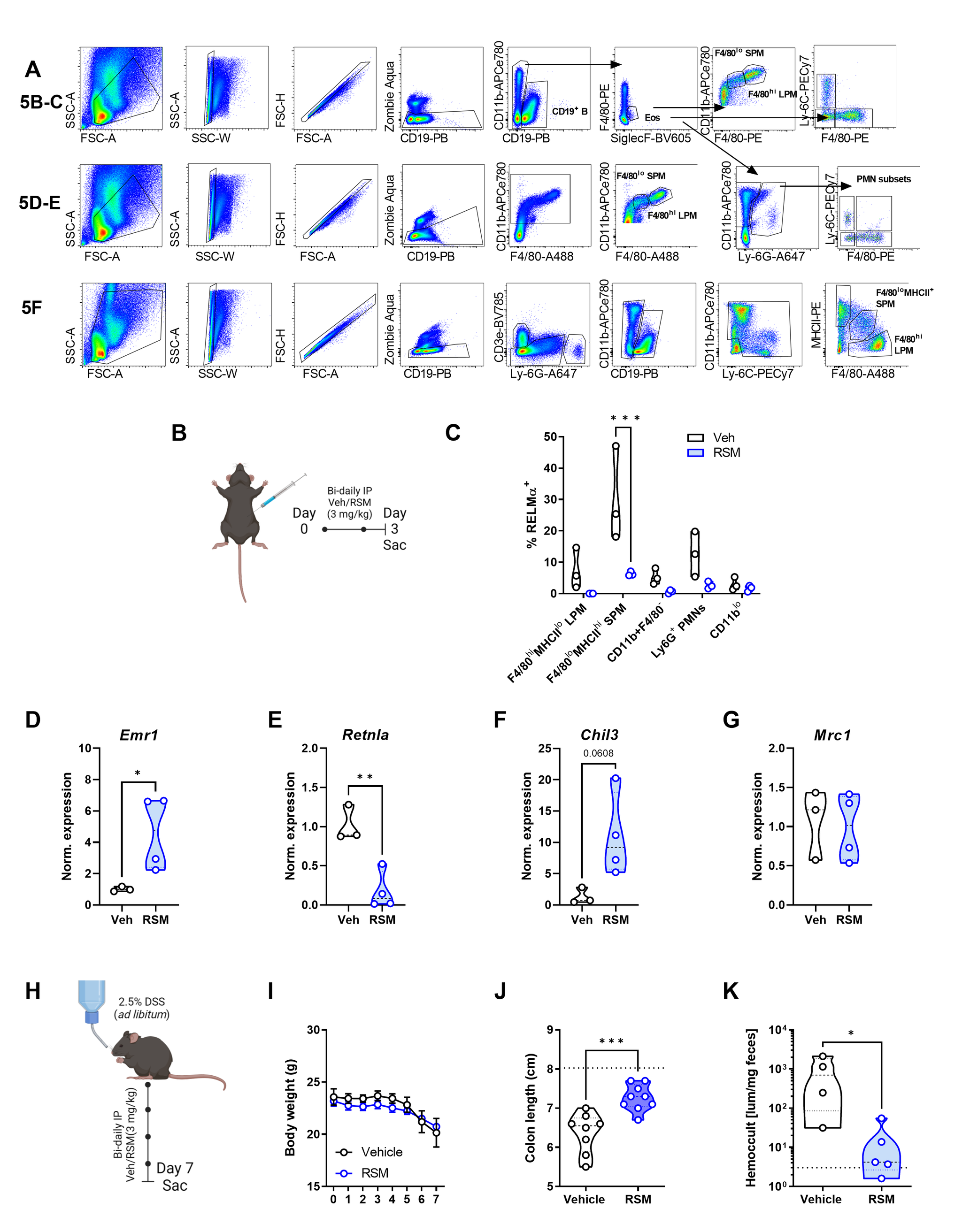

Supplement: S6 Fig — A) Gating strategies for peritoneal cells. B) Schematic of 3-day in vivo choline kinase inhibition. Mice were treated intraperitoneally with vehicle (40% DMSO in PBS) or RSM-932a (3 mg/kg) on day 0 and 2 and sacrificed on day 3. n = 3–4. C) Intracellular RELMα expression in live CD11b+ F480hiMHCIIlo, F480loMHCII+, F480−, Ly6G+ PMN, and CD11blo peritoneal cells. Two-way ANOVA with Šídák’s test for multiple comparisons (*** p < 0.001). D-G) Expression of Emr1 (Adgre1/F4/80), Retnla, Chil3, or Mrc1 in WAT. Unpaired t test (* p < 0.05, ** p < 0.01). H) Schematic of DSS-induced colitis. Mice were given 2–2.5% DSS in drinking water and treated with vehicle (40% DMSO in PBS) or RSM-932a (3 mg/kg) every other day for 6 days and sacrificed on day 7. I) Body weight was measured daily. n = 8–9, representing 2 independent experiments. J) Colon length on day 7. Reference naïve colon length in dashed line (8.025cm) from n = 4 mice. Unpaired t test (*** p < 0.001). K) Hemoccult detected in feces collected on day 6 normalized to weight. n = 4–5, representative of 2 experiments. Mann Whitney U test (* p < 0.05). Schematics were created using BioRender. (TIF) [file ppat.1011658.s006.tif]

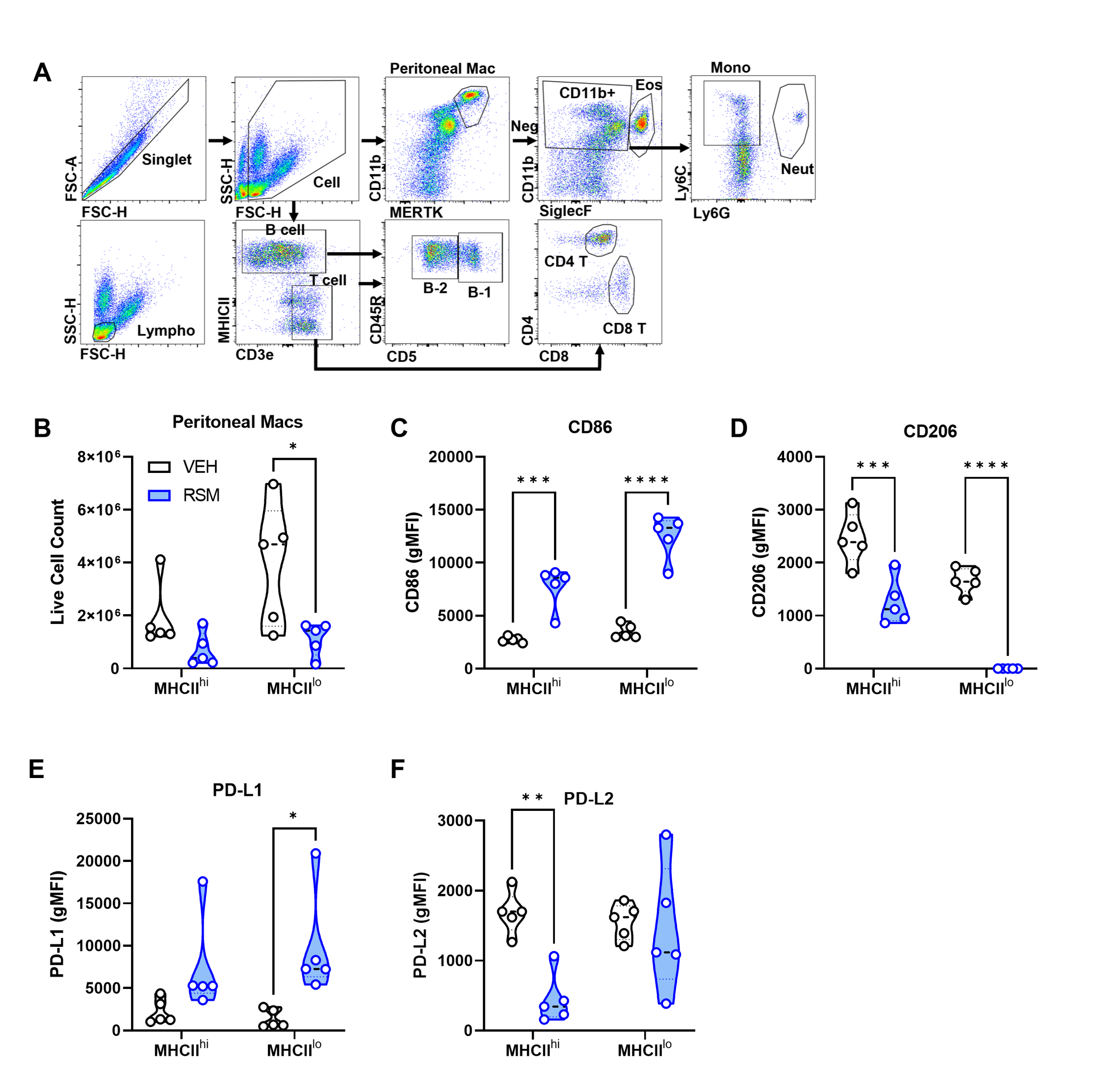

Supplement: S7 Fig — A) Gating strategy of peritoneal cell populations. B) Enumeration of MHCIIhi and MCHIIlo large and small peritoneal macrophages among live PECs. n = 4–5, representative of 2 experiments. Unpaired t test (** p < 0.01, **** p < 0.0001). C-F) CD86, D) CD206, E) PD-L1, or F) PD-L2 in MHCIIhi and MCHIIlo large and small peritoneal macrophages. Unpaired t test (*, p < 0.05, ** p < 0.01, *** p < 0.001, **** p < 0.0001). (TIF) [file ppat.1011658.s007.tif]

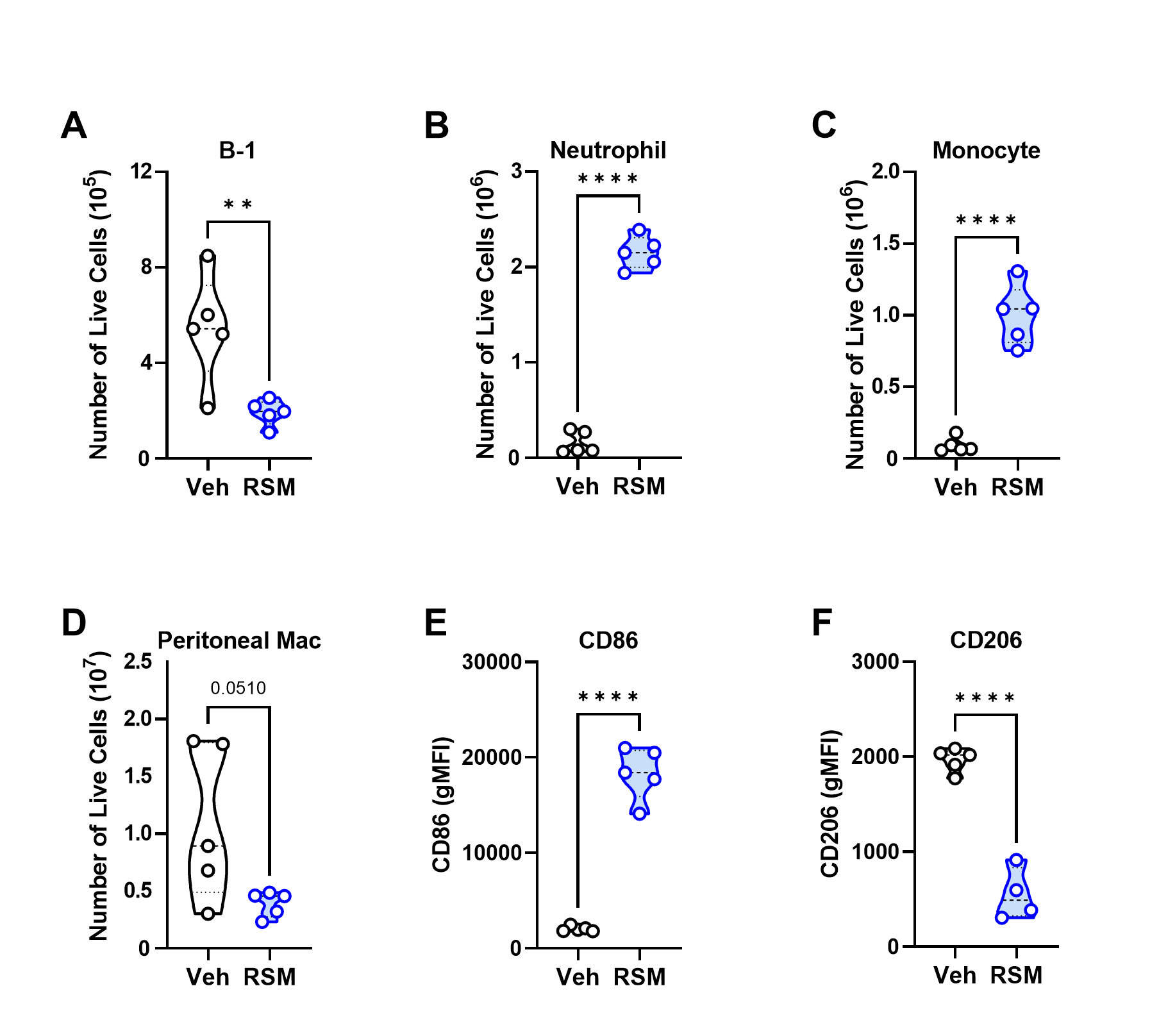

Supplement: S8 Fig — A-D) Enumeration of A) B-1 cells, B) monocytes, C) neutrophils, D) peritoneal macrophages among live PECs. n = 4–5. Unpaired t test (** p < 0.01, **** p < 0.0001). E-F) CD86, or G) CD206 expression (gMFI) on peritoneal macrophages. n = 4–5, representative of 2 experiments. Unpaired t test (**** p < 0.0001). (TIF) [file ppat.1011658.s008.tif]

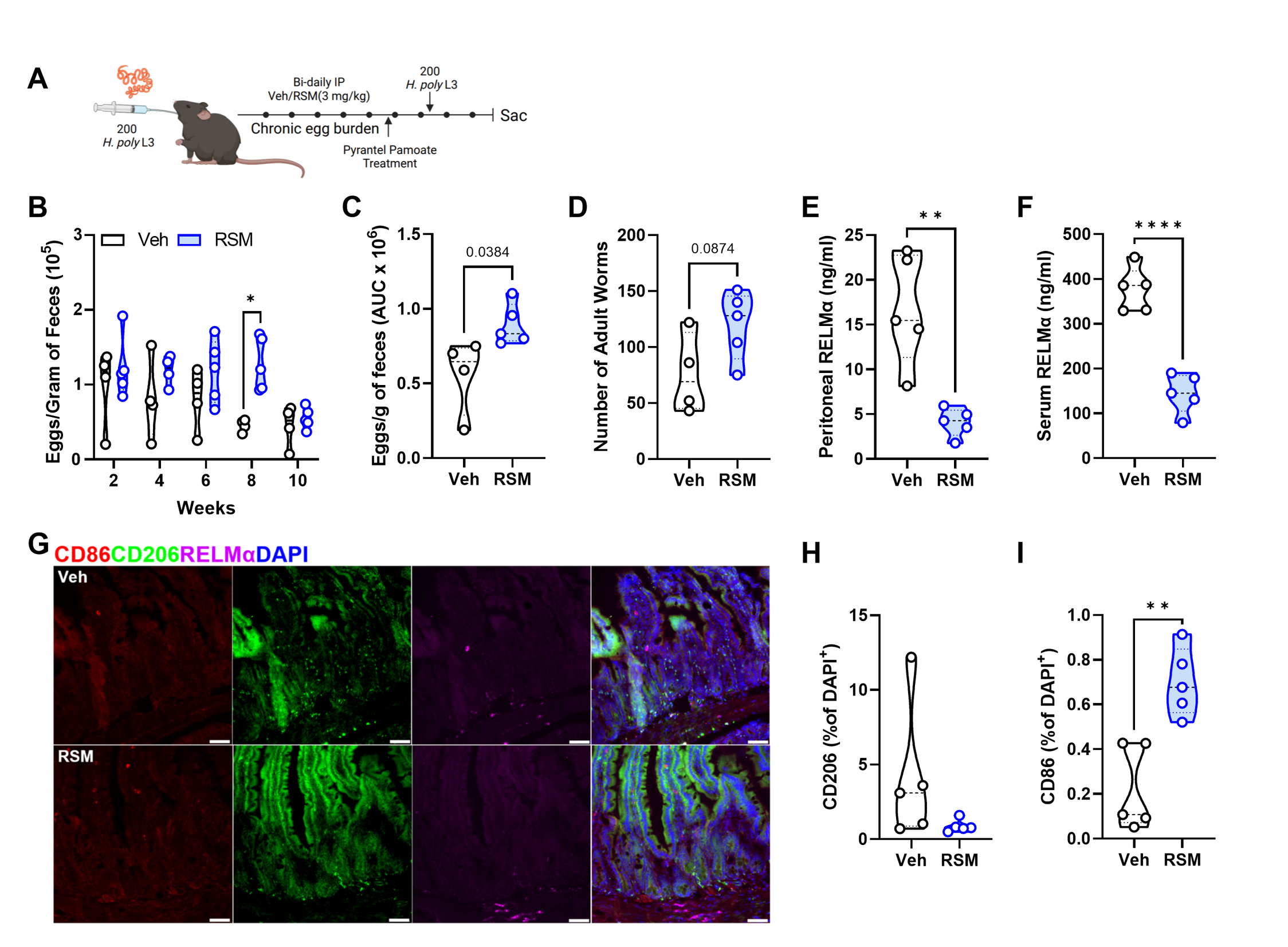

Supplement: S9 Fig — A) Schematic of secondary infection. Mice were infected with 200 H. polygyrus L3 larvae through oral gavage and intraperitoneally injected with vehicle (40% DMSO in PBS) or RSM-932a (3 mg/kg) every other day from day 2. Mice were treated with pyrantel pamoate (1 mg) at day 25, challenged with 200 H. polygyrus L3 larvae at day 42, and sacrificed at day 52 (D-G). For a long-term chronic infection (C), mice were infected with 200 H. polygyrus L3 larvae and intraperitoneally injected with vehicle (40% DMSO in PBS) or RSM-932a (3 mg/kg) two or three times per week. Then, mice were treated with pyrantel pamoate (1 mg) at day 93 and challenged with 200 H. polygyrus L3 larvae at day 107 and sacrificed at day 117. B-D) Eggs in feces were counted at multiple time points after long-term chronic infection, and the area under the curve (AUC) calculated (C). D) adult worms were isolated from the small intestine and enumerated at 52 DPI. n = 4–5 mice, representative of 2 experiments. Two-way ANOVA with Šídák’s test for multiple comparisons or unpaired t test (* p < 0.05). E-F) Detection of peritoneal fluid and F) serum RELMα by ELISA in naïve and H. polygyrus-infected mice. n = 3–5 per group, representative of 2 experiments. Unpaired t test (** p < 0.01, **** p < 0.0001). G-I) Representative immunofluorescent images of intestinal tissue stained for CD86, CD206, and RELMα against DAPI counterstain. Scale bar 50 μm. Quantification of CD206+ (H) or CD86+ (I) per DAPI+ cell. n = 5, representative of 2 experiments. Unpaired t test (** p < 0.01). Schematics were created using BioRender. (TIF) [file ppat.1011658.s009.tif]

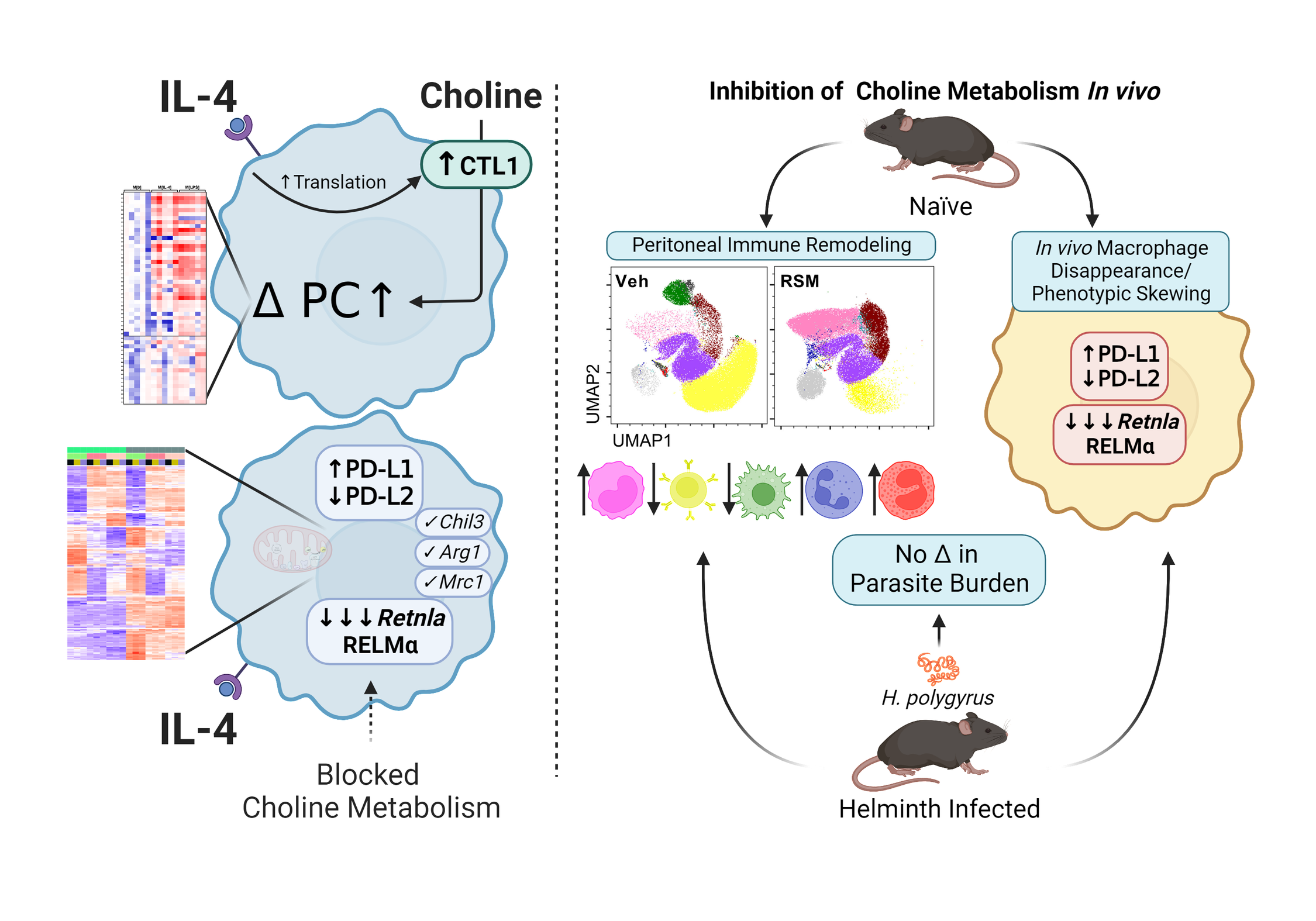

Supplement: S10 Fig — Created using BioRender. (TIF) [file ppat.1011658.s010.tif]
