# Supplementary material for: Choline metabolism underpins macrophage IL-4 polarization and RELMα up-regulation in helminth infection
Source: PLoS Pathog. 2023 Sep 25;19(9):e1011658. doi: 10.1371/journal.ppat.1011658 (PMC10553840; doi:10.1371/journal.ppat.1011658)
Supplement: Attachment — Submitted filename: Ghorbani and Kim et al. 2023- Response to reviews.docx [file ppat.1011658.s012.docx]

**Response to reviewer’s comments**

**Reviewer #1**:

1. It is not clear if choline metabolism is affecting the polarization process of M[IL-4] or cells that are already polarized and how specific this is to macrophage RELMa expression. It is important to clarify this aspect to support the manuscript’s conclusions.

a. In Fig. 3A-E and Fig. 4A-D, macrophages are pre-treated with HC3 or RMS to show effects of perturbing choline metabolism on polarization of M[IL-4]. In Fig. 3F, macrophages in the M[IL-4] state are treated to show effects on M[IL-4] phenotype. The authors should perform the assays in Fig. 3A-D and Fig. 4 in macrophages that are already M[IL-4] and should investigate IL-4Ra and RELMa protein expression.

To address the reviewer’s comments, we performed additional experiments that aimed to test the timeline in which inhibition choline metabolism is important for RELMa expression. Both choline uptake (HC3) and choline kinase (RSM) inhibitors were used for a total of 24 h, prior to, concurrent with or following 24 h of IL-4 polarization (Figure 2E). These results demonstrate that limiting choline metabolism in previously polarized macrophages is sufficient to suppress *Retnla* expression (a trend with HC3 and complete inhibition with RSM). At the transcript level, there were no changes in *IL4ra* expression.

b. The authors don’t show macrophage RELMa staining after H. polygyrus challenge (SFig. 5B-G) or in the peritoneal cavity (Fig. 7), making it hard to assess whether RSM treatment was impacting total macrophage populations or M[IL-4] macrophages, or just RELMa production in macrophages.

As per the reviewer’s suggestion, in this revised manuscript, we performed new staining of naive and D17 Hp-infected mice intestines with anti-F4/80 and RELMα antibodies. We show that RELMα-expressing macrophages were significantly decreased in the intestine of RSM-injected mice compared to vehicle-injected mice.

The authors could shed light on this issue by transferring monocytes from RSM- or vehicle- treated mice into IL-4-complex-treated animals and investigating M[IL-4] development and RELMa production from transferred cells.

While we agree with the reviewer that this would be an interesting experiment to conduct, we have plans to tackle these questions in future studies.

c. Krljanac et al. suggest that macrophage-derived RELMa supports host resistance to secondary N. brasiliensis infection (there are some caveats with the approaches used in these experiments, but the use of CD115iDTR with the RetnlaCre mice gets reasonably close to selectively deleting RELMa in macrophages). The authors should investigate whether RSM treatment and associated decreases in RELMa lead to impaired protection in N. brasiliensis secondary challenge.

Prior studies from our lab using RELMa KO bone marrow chimeras and whole body RELMa KO mice demonstrate that RELMa KO have equivalent or even better clearance of parasites in primary infection compared to WT mice, therefore, it is unlikely that these mice are more susceptible in secondary infection (we also have unpublished studies supporting this finding in secondary infection of RELMa KO mice). In the experiments reported here, we did not see differences in primary infection, therefore, we do not expect differences in secondary infection. This is also supported by a lack of difference in secondary responses with *H. polygyrus* infection that we report here in this study. On the other hand, in the Krljanac et al. study, all cells that expressed RELMα were deleted by apoptosis, likely causing inflammation, so any protective effectors they express would also be deleted (e.g., Arginase), therefore, this model is not specifically investigating RELMα deficiency. Based on these considerations, we do not expect differences in N. brasiliensis secondary challenge, and these experiments may not be relevant for this study. We discuss this paper in the manuscript.

2. There is no model to explain how inhibiting choline metabolism impacts M[IL-4] polarization. The authors show decreased pSTAT6 in macrophages pre-treated with choline metabolism inhibitors and then treated with IL-4 (Fig. 3E), but is there lower IL-4Ra expression on macrophages after choline metabolism inhibition? On page 8, the authors refer to data in Fig. 6 and SFig. 5 that show that the effects of choline metabolism on Retnla expression are IL-4-signaling-independent, but I can’t find these data (apologies if I missed them!). The authors don’t have to identify this mechanism – this seems beyond the scope of the current work to me - but as is, this issue is very murky in the text and should be discussed more clearly.

We appreciate the reviewer’s understanding. We have now determined that choline inhibition does not affect IL4Ra transcript expression, though this does not rule out post-transcriptional levels of regulation. The consistent decrease in pSTAT6 levels does not clarify the potential mechanism. The data previously shown in Figure 6 and S5 demonstrated a significant suppression of RELMα expression both in naïve and infected mice, which were extrapolated to represent with and without IL-4. We appreciate this is not entirely accurate and have modified the text accordingly.

**Reviewer #2**: Main comments:
a. The kinetics of choline metabolism inhibition in response to pharmacological drugs is quite puzzling. Why do you need 24h pre-incubation for seen an effect with a kinase inhibitor? This part would have gained to be strengthen by using some genetic models, e.g. siRNA-mediated KD or use of BMDMs from whole-body KO mice (if available).

We agree with the reviewer that genetic models would be an asset. While we have generated *Slc44a1* KO animals, we have not been able to validate the knockout. Moreover, we have collaborators who possess *Chka* heterozygous mice (Chka null mice are lethal in utero). Unfortunately, choline kinase activity in *Chka* heterozygous BMDM is upregulated to maintain haplosufficiency and fails to provide a suitable model for choline kinase inhibition (data not shown). These same collaborators are generating *Chka* LysM model; however, this is currently not available. We have reiterated in the discussion the limitations of our approaches. However, we maintain that having drugs inhibiting two independent targets of the CDP-choline pathway that show dose-dependence, provides strong evidence. As stated above in response to Reviewer #1, we attempted to address the reviewer’s comments around kinetics with additional experiments. We tested the timeline in which inhibition choline metabolism is important for RELMa expression. Both choline uptake (HC3) and choline kinase (RSM) inhibitors were used for a total of 24 h, prior to, concurrent with or following 24 h of IL-4 polarization (Figure 2E). While this does not clarify the mechanism, it does point to choline uptake and phosphorylation as being an important process for maintaining maximal *Retnla* expression.

b. The authors have used a knowledge-based selection of few alternatively-activated/M(IL4)/M2-like markers (notably Retlna/RELMalpha) to phenotype their macrophages in response to IL4 but would have gained to run at least few bulk RNAseq analyses in order to get an unbiased overview on the impact of choline metabolism inhibitors on macrophage identity.

We agree with the reviewer and chose to undertake an unbiased approach with bulk RNAseq analyses. As you will now see, this has added a significant amount to our manuscript and offered more clues as to how choline metabolism is linked to IL-4 responses. Interestingly, RNAseq analyses revealed an incredible downregulation of mitochondrial-encoded transcripts, which lead us to perform both extracellular flux analyses to map cellular metabolic pathways, as well as TEM to investigate subcellular architecture. While the results of these experiments are exceedingly clear, choline inhibition dramatically shifts cellular metabolism away from oxidative and toward glycolytic programs, whether there is a link to IL-4-related cytokine responses remains unknown.

c. The alterations of helminth-induced peritoneal immune cell phenotypes observed in response to pre-treatment with choline metabolism inhibitor are quite difficult to understand, mechanistically-speaking, and could for sure not be ascertained to a specific impact of the drug on macrophages, as it may be suggested in between lines. Altogether, although interesting/puzzling, this part is also a bit confusing and shifts the story flow beyond macrophage choline metabolism.

We agree that the shift in peritoneal cell populations, beyond changes in macrophages, may suggest that other cell subsets are directly affected by RSM treatment. Alternatively, since macrophages can promote these cell subsets (such as B1 cells and eosinophils), it is possible that the reduction in macrophages leads to the reduction in these cells. We now add these possibilities to the discussion.

**Reviewer #3:**

1. Figure 1B: Raw data of the western blots should be shown.

This has been added.

2. Figure 2: The authors indicate that the differences shown in panel B are significant. The authors should present the data to specifically indicate which lipids are significantly different in the bar graph.

The lipidomics data has been integrated into a new Figure 1, which addresses the reviewer’s concerns.

3. Figure 3: Are there any differences in macrophage viability with HC3 or RSM treatment before or after IL-4 stimulation? The plots in Figure 3E give this impression. This panel is also lacking labels for each plot.

Macrophage viability trends lower with HC3 or RSM, particularly with higher concentrations or longer timepoints (e.g., 48h). Our experimental approach to lift cells perhaps underestimates mortality, as the EDTA lifting is most effective following an initial PBS wash, where some non-adherent cells may have been removed. To this point, we arrived at treatments that minimize viability loss to non-significant levels by our flow cytometric analyses.

Despite differences in STAT6 activation, Retlna is the only M2 marker regulated by choline metabolism, suggesting that this pathway may not act, at least exclusively, on IL-4 dependent pathways to regulate Retlna. The authors should provide data to develop the underlying mechanism by which choline may regulate Relma but not other established STAT6-dependent genes.

The reviewer is quite right, the underlying mechanism(s) by which this distinct cellular profile arises, remains to be identified. Unfortunately, while we have identified interesting links to metabolism and cellular morphology that likely stem from choline incorporation into phospholipids, the exact mechanism will have to be part of future work. We hope the reviewer understands.

4. Figure 4: The authors suggest that the wound-healing effects are mediated by Relma, but provide no data to show actually demonstrating that Relma is active in this setting. While the use of Relma-/- macs would be ideal, the authors should at least compare these results to sups from non-polarized macs in the presence or absence of recombinant Relma treatment? Also the authors should cite the primary paper demonstrating the impact of Relma on wound healing (PMID: 26474656), not the review.

We now cite the original paper. We have discussed the limitations associated to our current approach, which is the associative and not causal link between impaired “wound healing” and lower RELMα levels in conditioned media.

5. Figure 5: This figure is unclear. Did these animals receive IL-4 complexes to induce Relma production? If not, why would the peritoneal macs be expressing so much Relma at baseline? This data is not consistent with previous papers showing minimal Relma production by macrophages at steady-state (see figure 2A in PMID: 24101381 and figure 1A in PMID: 21566158). Can the authors reconcile these published results from their data? In panels C, D and E, what UMAP clusters are you referring to? Macrophages are well-known to undergo a response known as the Macrophage Disappearance Reaction (MDR) to certain stimuli under settings of inflammation (PMID: 7884305; PMID: 31048328). This reaction also coincides with the recruitment of monocytes. Therefore, the phenotype observed in the peritoneum after i.p. injecting mice with RSM may be secondary to inflammatory effects mediated by drug injection. This issue needs clarification with additional controls and raw flow cytometry data showing Relma production by peritoneal macs should be provided to substantiate the graphs.

We thank the reviewer for their thorough assessment of the data. Upon review of the data, the reviewer was indeed correct and there was an error in our gating strategy that significantly skewed the designation of large and small peritoneal macrophage populations. Importantly, this gating was only affected for this naïve (no IL-4 complexes or *H*. *polygyrus*) experiment and has been revised. In the revised data set, we report RELMα expression in the SPM population, which is significantly lower upon choline inhibition with RSM. This is also consistent in our shorter 3-day protocol. Moreover, based on the reviewer’s suggestions, we also investigated the potential Macrophage Disappearance Reaction in this context. Using a recently published methodology, we collected and digested peritoneal aggregates separately from free-flowing lavage cells and also further subclassified macrophages with MHCII to better distinguish LPM from SPM. In addition, we performed trials to investigate potential other locations of macrophage egress but failed to locate LPMs in other organs including the liver, mesenteric lymph nodes, spleen, omentum, or thoracic LNs. Particularly the omentum and thoracic LNs were promising avenues from experiments we performed with intraperitoneal dye injections; however, the cells most prominently homing to these tissues are likely lymphocytes. We did however note from immunofluorescence analysis of omental milky spots and aggregate found floating or adhered to the peritoneal wall and noted strongly reduced RELMα expression. However, due to the complex nature of these experiments we were unable to incorporate them in this manuscript.

6. Figure 6: The regulation of Relma by choline is interesting, independent of its effects on resistance to helminth infection. Given the lack of impact that RSM treatment has on worm burden and the unconvincing microscopy results (panel F), I suggest strengthening the peritoneal dataset including, as mentioned above, FACS data on Relma production following H. polygyrus infection as previously shown (PMID: 28334040).

Although we did not perform intracellular RELMα staining of the peritoneal cell subset, we now perform rigorous and more expansive immunofluorescent staining of the intestinal tissue sections since this is the infection site. We now provide new data and quantification that demonstrated that F4/80+RELMa^+^ macrophages in the small intestine are significantly decreased with RSM treatment (see Figure 6K-M).

7. Figure 7: The same issues are relevant here as in Figure 5, in terms of macrophage disappearance, but more striking. In addition to the concern that the changes in the peritoneum are simply due to injecting an inflammatory agent independent of its biological activity, increased clarity as to whether the authors are examining M2 markers on tissue-resident macrophages, monocyte-derived macrophages or total macrophages needs to be provided.

We performed more in-depth flow analysis by gating MHCII^hi^ small peritoneal macrophages (derived from recently recruited blood monocytes) and MHCII^lo^ (derived earlier than MHCII^hi+^ macrophages) large peritoneal macrophages. In figure 7G, we showed that total peritoneal macrophages were downregulated when mice were injected with RSM-932A in both naive and infected condition. In a new analysis, the number and frequency of MHCII^hi^ and MHCII^lo^ peritoneal macrophages were decreased upon RSM injection and infection. Also, M1 markers such as CD86 and PD-L1 were significantly increased whereas M2 marker CD206 was upregulated in MHCII^hi^ and MHCII^lo^ macrophages in RSM-injected mice. Another M2 marker PD-L2 was decreased only in MHCII^hi^ macrophages in RSM-injected mice. This data is shown in Supplemental Figure S8.

**Minor Issues:**

**Reviewer #1**:

1. To better highlight key differences in the lipidomes of different macrophages, some panels from SFig. 2

could be moved to main Fig. 2.

We have reformatted these figures and included the most abundant lipid species in the main figure 1 of the revised manuscript.

2. Fig. 3E is missing the Vehicle, HC3, and RSM labels at the top of the flow plots.

Corrected and now shown as Figure 2G.

3. In SFig. 3E, on my screen, there’s no convincing pAKT S473 signal. This could be a resolution issue. If not, then these data could be removed without altering any conclusions.

This data has been removed.

4. The scratch wound data in Fig. 4D are out of place with the focus on RELMa. There is no data to show that effects of choline metabolism inhibition on the ability of macrophage-conditioned media to promote wound healing is RELMa-dependent (unless this is known from previous literature). If this effect is RELMa-dependent, this should be stated. If not RELMa-dependent, these findings are still interesting, they should just be presented in a different context.

There is a known effect of RELMα on wound healing (PMID 26474656; ref 57 here). This data is now shown in Supplemental Figure S3H.

5. The authors should explain the significance of increased CD86 expression in SFig. 5.

We provide an expanded discussion: “Moreover, RSM-932A increased CD86 expression in the intestine in the secondary *H. polygyrus* infection, which is a hallmark of M1-polarized macrophages (PMID: 12554797). CD86 in antigen-presenting cells provide costimulatory signals to activate T cells through CD28 and CTLA4 binding (PMID: 7513726 and 23470321). Previous studies reported that CD86 was also upregulated by helminth-derived peptide GK-1 in bone marrow-derived macrophages (PMID: 33049560). Thus, increased CD86 in macrophages may be an immunomodulatory effect of helminth-derived molecules to inhibit M2 macrophage polarization at the same time, and choline metabolism is likely responsible for downregulating CD86 upregulation.”

6. The supplemental figure legends should state how many experiments were performed.

These experiments were conducted once and are in the supplementary figures because they showed no difference or are supportive of the experiments in the main figures but not stand-alone. We have added this to the figure legends. These are long-term experiments with many mice, therefore, given that there were no differences, or they were not standalone, we believe it beyond the scope, and not a justified use of animals, to repeat. Nonetheless, we think it is helpful to provide as a supplement for the readers but will defer to the Editors and reviewers.

7. On page 6, as the authors discuss experiments with primary infection with H. polygyrus, they state that, “IL-4-polarized macrophages have been shown to play an important role in reducing helminth parasite burden; [29, 30]”. These references address the role of macrophages in N. brasiliensis secondary not H. polygyrus primary infection and they don’t address the role of RELMa. The authors later cite Anthony et al., which does establish a role for macrophages in protective immunity to H. polygyrus. In addition, it’s been shown that macrophage RELMa is important in reducing parasite burden in secondary N. brasiliensis infection (Krljanac et al.). The authors should clarify their discussion of the literature to make clear: 1) what was known previously, and 2) how their data fit with the literature.

We have now added the appropriate reference for 29, 30 and thank the reviewer for this correction. We have also expanded the discussion of the Krljanac paper, and prior studies with whole body RELMα KO mice.

8. The data shown in SFig. 5B-G do look as though there is some effect of RSM on protection, with trends to increased egg burdens, a significant difference at day 8, and a trend to increased adult worms. Are these observations consistent across replicate experiments?

The egg burden is increased at week 8 after infection, indicating that RSM-932A has an effect on the parasite burden, but only when it is injected chronically. While we conducted this experiment once, there are trends toward increased egg burden at all weeks. We now show the area under the curve for this chronic experiment where egg burden was quantified bi-weekly and there is a significantly higher overall egg burden in RSM treated mice (Supplemental Figure S7C). Of note, this effect was not observed in the other *H. polygyrus* infection experiments. We conclude that choline metabolism inhibition significantly reduces RELMα, but this is not sufficient to impact worm burden, however, can impact worm fertility. This may be partly explained by the fact that RSM does not inhibit other M2 effectors such as Arginase. We discuss this in the revised text.

9. The gut N. brasiliensis burdens in SFig. 7D are quite low for day 6. Can the authors comment?

We maintain N. brasiliensis life cycle in rats, and they are less virulent compared to mouse-adapted N. brasiliensis, and we typically see lower intestinal burdens than other groups. This is consistent in all the WT mice maintained in our vivarium.

10. In SFig. 7, the authors show a decrease in RELMa staining from lung epithelial cells in mice treated with RSM and infected with N. brasiliensis. What about macrophages?

We stained naive and D6 Nb-infected mice lungs with anti-F4/80 and RELMα antibodies, and RELMα expressing macrophages were significantly decreased in the lung of RSM-932A-injected mice when compared to vehicle-injected mice (see Figure 8)

**Reviewer #2**: Minor comments:

- Couple of typos should be corrected throughout the manuscript, including some in the abstract (“by injection IL-4”, “no difference intestinal worm”)

Corrected.

- Some rephrasing are also required, e.g. “using similar but still differential study designs”

Corrected.

- Please clarify the rationale for measuring Ser473-pAkt

This blot has been removed from the revised manuscript.

- The authors claim that “in vivo inhibition of choline metabolism restructures endogenous tissue-resident macrophage phenotype”. However, it is unclear how they define “tissue-residency”. MHC-II and/or TIM-4 expression coupled to F4/80 is generally used to discriminate newly recruited monocyte-derived macrophages versus long-lasting tissue-resident macrophage populations.

The new data in Figure 5 and Supplemental Figure S6 was generated using MHCII as a marker.

- Statistical analyses on Figure 3F are missing

This experiment has been repeated and now shows proper and relevant statistical analyses.
