# Supplementary material for: Choline metabolism underpins macrophage IL-4 polarization and RELMα up-regulation in helminth infection
Source: PLoS Pathog. 2023 Sep 25;19(9):e1011658. doi: 10.1371/journal.ppat.1011658 (PMC10553840; doi:10.1371/journal.ppat.1011658)
Supplement: Attachment — Submitted filename: Ghorbani and Kim et al. 2023- Response to reviews.docx [file ppat.1011658.s013.docx]

**Response to reviewer’s and editor’s comments:**

**From the editor:**

One reviewer has raised an important issue that was not addressed from the first review, and that is the number of experimental replicates in the data in the supplemental experiments (See reviewer 1). I am requesting that this experiment be removed from the manuscript if a replicate experiment can not be added that has the same trends and significance. I agree with the Reviewer that the standard in the field is a minimum of duplication, but ideally 3 biologically independent experiments. There is also a minor point in figure 4 that needs to be fixed for clarity of readers listed by Reviewer 2.

We have removed the experiment in question, previously shown in Figure 8. Moreover, we have changed the axis as requested by reviewer #2.

**Reviewer #1:** My original Minor Point #7 requested that the number of replicate experiments performed for all supplemental figures be added to the legends. In the authors' response, they indicated that these experiments were only performed once, as supportive of the main findings and not, "stand alone."

I appreciate that these are lengthy experiments and difficult to do multiple times. However, presenting data from experiments only performed once is not in line with standards in the field. Independent, replicate experiments should be performed at the very least twice, and ideally, 3 times. If these experiments cannot be repeated, the data should not be presented, in my opinion. Even if the data are confirmatory of other findings and in the supplement, I feel that the standards for rigor should be maintained.

I will defer final judgment on that to the Editorial team. However, if the Editorial team feels that inclusion of data from single experiments is acceptable, I request that this fact be very clearly acknowledged in the legends, and in the text itself as well.

We agree with the reviewer and have removed the experiment in question, previously shown in Figure 8. We have also clarified number of experiments in the figure legends where appropriate.

**Reviewer #2:** The y-axis scale of the Seahorse data should be similar for allowing easy comparisons (Fig 4C&E)

Thank you for the suggestion, we have made the change. This allows for a nice comparison and visualization of IL-4 stimulation of oxidative metabolism.
